# A Systematic Review of Mobile Applications to Support Individuals With Cerebral Palsy and Their Caregivers

**DOI:** 10.7759/cureus.93848

**Published:** 2025-10-04

**Authors:** Md Razeen Ashraf Hussain, Syeda Sabrina Easmin Shaba, Israt Jahan, Mahmudul Hassan Al Imam, Mohammad Muhit, E Bunthen, Iona Novak, Nadia Badawi, Gulam Khandaker

**Affiliations:** 1 Research and Development, CSF Global, Dhaka, BGD; 2 Graduate School of Biomedical and Health Sciences, Hiroshima University, Higashihiroshima, JPN; 3 School of Health, Medical and Applied Sciences, Central Queensland University, Rockhampton, AUS; 4 Central Queensland Public Health Unit, Central Queensland Hospital and Health Service, Rockhampton, AUS; 5 Research and Public Health, CSF Global, Dhaka, BGD; 6 National Payment Certification Agency, Ministry of Economic and Finance, Phnom Penh, KHM; 7 Faculty of Medicine and Health, University of Sydney, Sydney, AUS; 8 Child and Adolescent Health, Cerebral Palsy Alliance Research Institute, Brain and Mind Centre, University of Sydney, Camperdown, AUS

**Keywords:** apps, cerebral palsy, cp, mobile application, review, systematic review

## Abstract

This study aims to systematically review the effect of mobile applications (apps) in supporting individuals with cerebral palsy (CP) and their caregivers. Five databases were searched for articles published between 2013 and 2023. Included studies were original with full available text that assessed the effectiveness of mobile apps to support the daily life of individuals with CP and their caregivers. The Risk Of Bias In Non-randomised Studies - of Interventions (ROBINS-I) tool was used to assess the risk of bias, and quality of evidence was assessed using the Grading of Recommendations Assessment, Development and Evaluation (GRADE) approach. Six studies, encompassing 91 individuals with CP, were included. Included studies were mostly experimental (3/6, 50.0%). Predominantly focused on children with CP, the studies covered various areas, such as assistance with speech impairment, intervention mapping with gamification, athletics, relaxation, and educational apps for individuals with CP. Among all, one study focused on caregivers. Out of six studies, two were found to be serious (33.3%), and four (66.7%) had a moderate risk of bias. Quality assessments revealed that grades were low (4/6, 66.7%) and very low quality (2/6, 33.3%). The limited available studies indicate the need for future research on the potential of integrating technological solutions, such as mobile apps, in addressing various facets of management and care of individuals with CP.

## Introduction and background

Cerebral palsy (CP) is a group of disorders that affect a person's ability to move and maintain balance and posture. CP is the most common movement disorder in children [1]. CP is caused by abnormal brain development or damage to the developing brain that affects its ability to control muscles, but the severity and clinical features vary from individual to individual [1,2]. In high-income countries (HICs), the estimated birth prevalence of CP is 1.6 per 1000 live births, whereas in low-and middle-income countries (LMICs) this rate is substantially higher [3].

The motor type, topography, and associated impairments can vary widely [4]. Although CP is a non-progressive disorder, the motor function and clinical severity can deteriorate in adolescence in the severe motor types. Depending on which area of the brain is affected, CP can cause muscle stiffness (spasticity), twisting postures (dyskinesias), balance and coordination problems (ataxia), and reduced tone (hypotonia), all of which cause difficulties with movement [5]. There is no cure for CP, but early intervention and rehabilitation can improve the lives of those affected [6,7]. As a result, early diagnosis of CP is important for the health of children and their families [6]. Traditionally, delays in rolling, sitting, standing, and walking might trigger investigations for CP, but now an earlier diagnosis is possible using three tests outlined in a clinical guideline [7,8].

Technology advancements have revolutionized healthcare, enabling personalized, participatory, and proactive services [9,10]. Various mobile applications (apps) and medical software contributed to enormous daily life care and time management to clinical decision-making at the point of care for various conditions, including CP [11]. Previously reliant on basic therapies, CP care now includes advanced mobility aids, communication devices, and therapeutic tools such as virtual reality and robotic therapy [12]. Wearable sensors and telemedicine enable continuous, personalized care. These technologies significantly increase motor skills, communication, and independence, especially in resource-limited environments [12]. Additionally, rigorous evaluation of mobile apps played a huge modifying behavior to promote health and manage disease, including different disabilities such as CP [9,11]. Mobile apps are taking on a more significant role in CP care through increased therapy, communication, and caregiver access. However, data on their effectiveness and quality are scattered.

This study aims to systematically review published effectiveness research on mobile apps designed to support individuals with CP and their caregivers.

## Review

Methodology

Protocol Registration and Study Guidelines

This systematic review was conducted following the 2020 Preferred Reporting Items for Systematic Reviews and Meta-Analysis (PRISMA) guidelines [13]. The protocol was registered in the International Prospective Register of Systematic Reviews (PROSPERO) (registration number: CRD42023485027). 

Literature Search

The literature was systematically searched on five electronic databases: Web of Science, Scopus, CINAHL (Cumulative Index to Nursing & Allied Health Literature), Embase, and PubMed. The PICO (population, intervention, comparator, and outcome) search term keywords were: Cerebral palsy (population, Mobile applications, mobile app, App, smartphone (Intervention), with any comparator or outcome accepted. The literature search was filtered to include articles published between 2013 and September 2023. The search was modified to match the particular structure of each database (Table 1). 

**Table 1:** Search strategy online databases CINAHL: Cumulative Index to Nursing & Allied Health Literature

No.	Data Bases	Keys Words	Number
1	PubMed	("Cerebral Palsy"[MeSH] OR "CP Cerebral Palsy") AND (("Mobile Application"[MeSH] OR "Mobile Apps"[MeSH] OR "Apps"[Mesh] OR "Apps, Mobile" [Mesh] OR "Smartphone"[Mesh]))	43
2	Web of Science	(TS="Cerebral Palsy " OR TS= CP") AND ((TS= Mobile Application OR TS= Mobile Apps OR TS= Apps OR TS=e Apps, Mobile OR TS= Smartphone)	30
3	Scopus	ALL (Cerebral Palsy) OR ALL (CP) AND ALL (Mobile Application) OR ALL (Mobile Apps) OR ALL (Apps) OR ALL (Smartphone)	124
4	CINAHL	("Cerebral Palsy" OR "CP Cerebral Palsy") AND ("Mobile Application" OR "Mobile Apps" OR "Apps, mobile" OR "Smartphone")	42
5	Embase	('cerebral palsy'/exp OR 'cerebral palsy' OR (cerebral AND ('palsy'/exp OR palsy))) AND ('mobile application'/exp OR 'mobile application' OR (mobile AND ('application'/exp OR application)) OR 'mobile app'/exp OR 'mobile app' OR (mobile AND ('app'/exp OR app)) OR 'app'/exp OR app OR 'smart phone'/exp OR 'smart phone' OR (('smart'/exp OR smart) AND phone))	35
Total			274

Study Selection and Strategy

The qualifying criteria for this systematic review were developed to guarantee that articles relevant to the study issue were chosen with acceptable methodological rigor. Studies were included if they included participants of any age and geographic region who had CP or were their caregivers. To be qualified, studies were required to study the impacts of mobile apps and given data on critical outcomes, such as their effectiveness in enhancing individual quality of life (QoL). We only examined papers with rigorous research approaches, such as randomized controlled trials, experimental studies, and observational studies. Furthermore, only peer-reviewed publications published between 2013 and 2023 were included to keep the emphasis on current, high-quality research. The studies included were (i) original articles, (ii) studies that reported the effectiveness of mobile apps to support individuals with CP and their caregivers in daily life activities and improvement, and (iii) full-text available. Studies without full text, pertaining to the validation of mobile apps and addressing physical disabilities other than CP, articles focused on robotics, telerehabilitation, and devices other than mobile apps were excluded. We limited ourselves to peer-reviewed publications to ensure rigor, while validation studies were excluded, as our focus was on applied use rather than tool development.

Screening of the titles and review of the abstracts were completed by two independent reviewers (MRAH and BE) to confirm a match with the inclusion criteria. Relevant articles that met the primary inclusion criteria during title screening and abstract review were selected for full-text review. Two independent reviewers (MRAH and BE) conducted an in-depth assessment of the full texts of the included studies, and studies that met the eligibility criteria were included in the study. In case of any disagreement between reviewers on inclusion, a third review (GK) was consulted, and discussions were held until a consensus was reached. 

Data Extraction and Management

Rayyan, a free web and mobile application developed by Qatar Computer Research Institute, was used to manage the review via a semi-automated process, including initial screening of both abstracts and titles based on predefined inclusion and exclusion criteria [14]. Rayyan allows for blinded, independent assessments by multiple reviewers, minimizing bias. This tool helped streamline the process by automatically flagging studies based on keywords and facilitating real-time collaboration, ultimately enhancing the efficiency and transparency of the screening process. 

Inter-rater reliability was not formally calculated because, despite all the disagreements, a consensus was arrived at through discussion. Conflicts between reviewers (MRAH and EB) were resolved through discussion or by a third reviewer (GK). EndNote X9 (Clarivate Plc, London, United Kingdom) was employed to manage citations. Publications were distributed equally between the reviewers (MRAH and BE), and each researcher read each paper completely to determine its topic, extract its limitations and future recommendations, write a summary, and record this information in a Microsoft Excel spreadsheet (Microsoft Corporation, Redmond, Washington, United States.

A standard data extraction template (Excel sheet) was developed a priori by the investigators, piloted with a few studies, and refined before starting the full data extraction. The following data were extracted from individual studies, (i) bibliographic information, (ii) aim/objective of the study, (iii) sample size and demographic characteristics of the study participants (iv) intervention of the application (v) outcomes, benefits and indicators used and (vi) numerical/ narrative findings related to outcome measure/ results (if available). The collected data were assessed for heterogeneity before conducting the meta-analysis. The Cochran’s Q test was used to determine heterogeneity, and the I^2^ index was used to quantify it. When the p-value of the Cochran’s Q test was less than 0.05, heterogeneity was considered significant. I^2^ of ≥ 50% was considered as substantial heterogeneity.

Risk of Bias Assessment and Quality Assessment

The Risk of Bias in Non-randomized Studies - of Interventions (ROBINS-I) tool was used to assess the risk of bias of non-randomized studies of interventions [15]. Our review focused on assessing real-world application and implementation of mobile apps in CP care and also evaluating intervention efficacy. Therefore, we applied the ROBINS-I tool consistently across studies to maintain comparability in risk-of-bias assessment for applied usage. Two reviewers (MRAH and BE) independently assessed the quality of the included articles, and conflicts were solved through discussion with a third reviewer (GK). The ROBINS-I tool consists of seven domains, and each domain can be judged as having a low, moderate, serious, or critical risk of bias or no information. If at least one domain was assessed to be at serious risk of bias, the study was defined as a study with a severe risk of bias.

For the quality assessment, the quality of each included study was assessed separately by two reviewers (MRAH & BE) following the Grading of Recommendations Assessment, Development and Evaluation (GRADE) framework [16]. GRADE offers a widely accepted method to grade evidence quality and determine the strength of recommendations in patient management guidelines. This systematic approach enhances scrutiny and transparency in decision-making. The quality of evidence was classified into four categories (high, moderate, low, and very low quality) based on issues such as bias, inconsistency, indirectness, imprecision, and publication bias. Two independent reviewers (MRAH and BE) performed the quality assessment process, and any dispute was discussed. If no decision was obtained, the third reviewer (GK) was consulted to reach a consensus. The GRADE technique offered a transparent and systematic framework for measuring confidence in effect estimates, ensuring that the review's conclusions were robust.

Results

**Figure 1:**
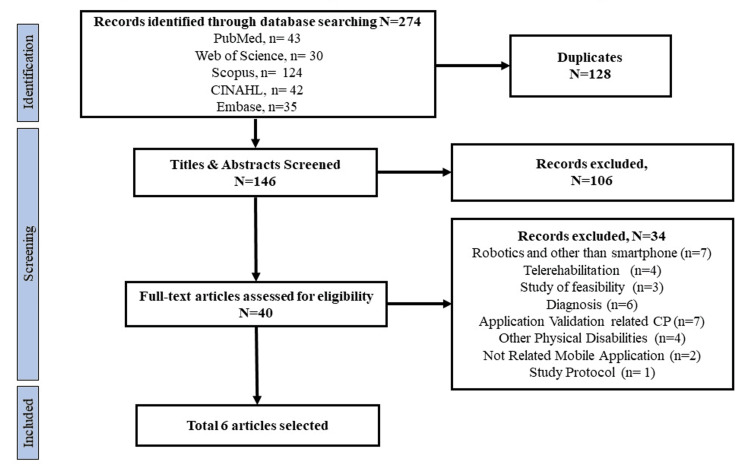
PRISMA flow chart showing the selection process PRISMA: Preferred Reporting Items for Systematic Reviews and Meta-Analysis

In total, we identified and screened 274 potential studies by conducting a comprehensive search in the selected databases. After removing 128 duplicate articles, 146 papers were considered for the title and abstract evaluation. In the screening stage of selection, 106 studies were excluded according to the selection criteria. A total of 40 articles underwent full-text review, and at this stage, another 34 were excluded based on the exclusion criteria. Finally, we included six articles meeting the inclusion criteria and conducted data extraction [17-22]. Figure 1 presents the PRISMA flow diagram of the detailed selection process of the studies.

Additionally, during the eligibility assessment and screening of the full text of the study, we identified an additional six studies that had special merit with regard to children with CP; however, they didn’t match our selection criteria as they were focused on assessment, not intervention. These six studies focused on the validation and early detection of CP and self-care management of apps related to daily life improvement and diagnosis of individuals with CP [23-27]. 

Our assessment of the collected data revealed substantial heterogeneity, as indicated by I² values consistently above the threshold of 40%. Specifically, the Cochran’s Q test results showed that heterogeneity exceeded 50%, categorizing it as substantial. Due to this high level of heterogeneity, a meta-analysis could not be conducted. The results were varied, classified with various interventions on sub-groups, so it was not possible to evaluate method and clinical heterogeneity by means of sub-group analysis or meta-regression modelling. However, we conducted quality assessment and risk of bias assessment separately, which provided an overview of bias and details about the study.

Characteristics of the Included Studies

This review included two studies from Australia, two from Brazil, one from Malaysia, and one from Iran. Five out of six of the study participants were individuals with CP [18-22]. Among the included studies, two focused on assistance with speech impairment [18,21], one on neurodevelopment [20], one on professional footballers with CP [17], one on relaxation intervention for individuals with CP [22], and one related to educational apps for individuals with CP [19]. All of the included studies were designed for individuals with CP, with a focus on children. However, there was one study specifically designed for caregivers of individuals with CP [19]. All the articles were original research published in peer-reviewed journals. In terms of study design, three out of six studies were experimental studies [17,19,22], and the remaining included one cross-sectional study [20], one case study [21], and one user-based evaluation study [18]. Table 2 presents the characteristics of the included studies.

**Table 2:** Main characteristics of the included studies in the systematic review

Title	Study Design	Study Participants	Number of participants	GRADE
Coswig et al. [17]	Experimental cohort study	Individual with CP including athletes and footballer	Football players including individual with CP, N=40	Low
Silva et al. [18]	User based evaluation study	Individuals with CP and speech impairments	Individual with CP, N=20	Low
Ghazisaeedi et al. [19]	Experimental pre-post study	Children with CP and their caregivers	Individual with CP, N=17	Low
Johnson et al. [20]	Cross Sectional Study	Individual with neurodevelopment disabilities	Individual with CP N=3, Others, N=1	Very Low
Zamin et al. [21]	Case study	Child Individual with CP specifically with speech impairment	Individual with CP, N=1	Very Low
Ostojic et al. [22]	Mixed methods, including experimental pre-post study	Children with CP	Individual Spastic with CP and dyskinesia with CP, N=10	Low

Key Findings

The studies focused on various apps designed to support different aspects of CP management, encompassing sign language for those with neurodevelopmental disabilities [21], enhancing accessibility and communication for a broader range of individuals [18], improving QoL with behavior changing technique [20], pain and relaxation management [22], potentially aiding in athletic development among individuals with CP [17], and evaluating caregivers' educational knowledge of CP management [19]. Zamin et al. implemented a mobile-based Augmentative and Alternative Communication (AAC) tool for non-verbal children with CP, resulting in successful communication with parents and caregivers, eliminating the necessity for sign language and improving speech and language development [21]. Johnson et al. developed a user-centered therapy prescription app for 6-12-year-old children with neurodevelopmental disabilities, employing intervention mapping and gamified design, resulting in enhanced apps through a systematic, flexible approach that considered user-centered design, behavior change via the intervention mapping (IM) process, and engagement with gamification [20]. Silva et al. implemented the AACVOX app, a mobile technology-based tool for AAC, tailored to the motor limitations of individuals with cerebral palsy, resulting in the development of user-friendly software with a customizable interface to facilitate usage for people with varying levels of motor disabilities [18]. Coswig et al. evaluated the reliability of the MyJump2 app for assessing vertical jump performance in professional CP Football athletes, revealing its higher effectiveness compared to the contact mat in measuring jump height and flight time for both squat jump and countermovement jump [17]. Ghazisaeedi et al. assessed the impact of an educational mobile application on caregivers of children with CP and found that training through novel technologies, such as a mobile app, enhances caregivers' knowledge about the daily care of children with CP [19]. Ostojic et al. reported that the acceptability of biofeedback-assisted relaxation training through the BrightHearts app for chronic pain management in children with CP might be beneficial as part of a multimodal approach, impacting pain and anxiety intensity measured on a numerical rating scale [22]. The interventions and major outcomes of the included studies are discussed in Table 3.

**Table 3:** Major findings of the included studies in the systematic review

Study	Developed Application	Application Type	Intervention	Major outcome
Coswig et al. [17]	MyJump2	High speed camera-based measurement app	Evaluated the vertical jump performance such as countermovement jump and squat jump in professional CP Football athletes to assess neuromuscular status	The MyJump2 app demonstrated high reliability in measuring jump height and flight time for squat jump and countermovement jump in elite CP Football athletes that indicated the potential utility for comprehensive jump performance analysis
Silva et al. [18]	AACVOX	Augmentative and Alternative Communication (AAC) app	Implemented AACVOX, a mobile tool for augmentative and alternative communication tailored to CP motor limitations	The outcome of AACVOX was user-friendly and customizable interface, significantly contributes to enhance accessibility and usability for individuals with varying degrees of motor disabilities.
Ghazisaeedi et al. [19]	Mobile Health and E-learning application (Prototype)	E-learning based educational app	Utilized of mobile app with several educational modules significantly enhances the knowledge of caregivers for children with CP	Implemented educational Android-based application significantly improved caregivers' knowledge of appropriate daily care for children with CP and additionally enhanced their ability to access supplement to existing treatment and rehabilitation practices for children with CP.
Johnson et al. [20]	Zingo App (Prototype)	User-centered intervention mapping and gamified app	Developed a user-centered therapy app for 6-12-year-olds with neurodevelopmental disabilities, using intervention mapping and gamified design embedded with behavior changing techniques theory	Developed app for prescription of home and school therapy activity programs for children with disabilities, incorporating theory-based behavior change techniques was effective and an engaging and fun app for the targeted population
Zamin et al. [21]	Make me Speak	Augmentative and Alternative Communication (AAC) app	Implemented a mobile AAC tool for non-verbal children with Cerebral Palsy who could communicate significantly with their parents and carers without using any sign language	App assisted successful communication, eliminating sign language needs, and enhancing speech and language development. Additionally, assisted to overcome the difficulty of speaking and increase the independency level.
Ostojic et al. [22]	BrightHearts	Control and Analyzing Biofeedback assisted relaxation training device	Biofeedback-assisted relaxation training (BART) with BrightHearts for chronic pain management in children with CP	Demonstrated the acceptability of BART for the management of chronic pain in children and adolescents with CP but no evidence of changes pain intensity or anxiety intensity scores between baseline and conclusion of the intervention from the self-report of participants.

Studies with Special Merit

This review found an additional five articles focused on the validation, early detection of CP, and self-care management of mobile apps for CP individuals and caregivers during the screening process. Those studies had not come under our inclusion criteria, but were related to CP management. Out of five, three studies were about the validation of mobile apps, including smartphone-based goniometer apps to measure range of motion (ROM) in hip abduction [27], migration percentage on hip surveillance [26], and femoral anteversion in adults using standard radiographs [24]. Two studies focused on the early detection of CP using mobile apps, specifically utilizing General Movements Assessment (GMA): Baby Moves [25] and NeuroMotion [23]. Table 4 includes the details of these studies.

**Table 4:** Studies with special merit related to individuals with CP

Study	Participants	Target Participants	Intevention	Results
Svensson at el. [23]	n= 37 CP Individual, n=0	Early detection of CP through perinatal brain injuries and GMA	Usability of NeuroMotion app regarding film quality and user experience and to assess the inter-rater reliability of GMA in a neonatal risk group	App generated high-quality technical videos, positive experience of users and the level of agreement between on-site assessors and the expert in GMA was high.
Lee at al. [24]	n= 94 CP Individual, n=12	Individuals with CP and with postoperative evaluation of femoral torsion	Validation and use of a mobile application that can reconstruct a three-dimensional model of the femur from conventional radiographs for adults	Application demonstrated excellent validity and reliability in measuring femoral anteversion in adults using standard radiographs compared to CT, offering a cost-effective and accessible option for clinical settings.
Kwong et al. [25]	n= 451 CP Individual, n=0	Extremely Preterm (EP) or extremely low birthweight (ELBW) infants	Assess the engagement with Baby Moves app on General Movement assessment amongst high- and low-risk infants’ families.	Most parents in this study successfully used Baby Moves to capture infant movements for remote GMA. Families of lower sociodemographic status used Baby Moves less
Kulkarni et al. [26]	n= 37 CP Individual, n=0	children with CP for Hip surveillance	Assessed the migration percentage o and calculated the mean absolute error in comparison to the reference standard obtained on a radiology workstation.	Accurate and reliable means of measuring migration percentage on hip surveillance radiographs which allow evaluating hip displacement in children with CP.
Johansen et al. [27]	n= 50 CP Individual, n=50	Individuals with cerebral palsy	Used both a traditional universal goniometer and a smartphone-based goniometer application to measure range of motion in hip abduction, popliteal angle, and ankle dorsiflexion	A photography-based goniometer can be a reliable and valid tool when measuring range of motion in children with cerebral palsy.

Risk of Bias and Quality Assessment

**Figure 2:**
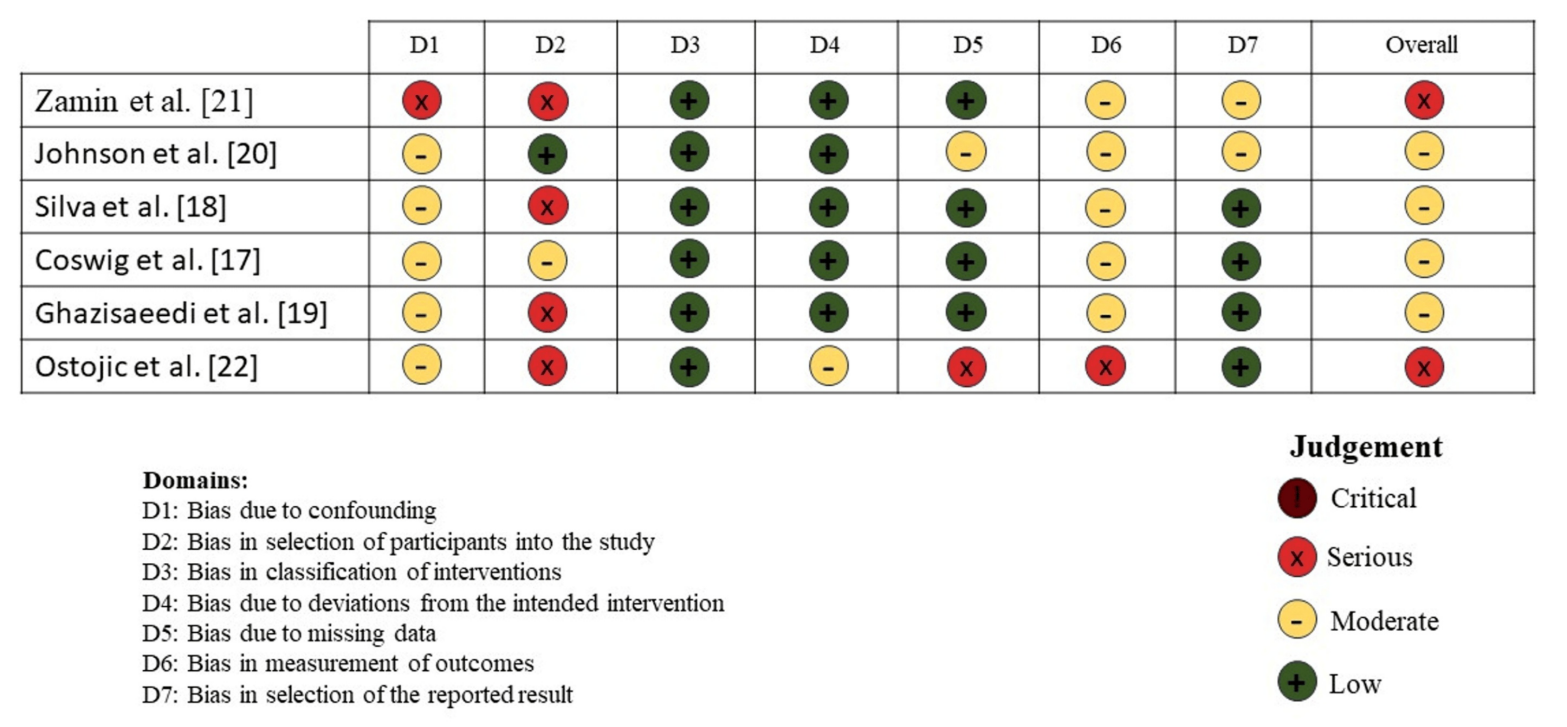
Risk of bias assessment ROBIN-I traffic light plot Each study is assessed across seven bias domains, covering pre-intervention, intervention, and post-intervention phases. The color-coded circles represent the level of bias: green for low risk, yellow for moderate risk, red for serious risk, and brown for critical risk. References: [17-22] ROBINS-I: Risk Of Bias In Non-randomised Studies - of Interventions

The ROBINS-I tool was used to assess individual risks of bias. Two studies were rated as serious risk of bias (33.3%) [24,25]. Overall risk of bias was found in four studies [20-23] out of six (66.7%) as moderate risk of bias. Each outcome of the ROBIN-I traffic signaling light plot is shown in Figure 2, and detailed information on the answers to the signaling questions is provided in the Appendices. All studies included in this review used a non-probability sampling technique (e.g., participants with CP were deliberately chosen). Randomization was not used because it is ethically unacceptable to carry out specific interventions solely for research purposes. The GRADE employed in our systematic review, where most of the included studies were of low quality (4/6, 66.7%), with two study rated as very low quality (2/6, 33.33%) (Table 1). The rationale of judgment of included studies on the ROBIN-I assessment is given in the Appendices.

Discussion

To the best of our knowledge, this is the first systematic review focusing on the effectiveness of mobile apps related to the daily life improvement of CP individuals and their caregivers. Our study found that all the apps could play a vital role on daily life support and improvement of CP individuals on different aspects such as speech impairment, neurodevelopment disorders, neuromuscular status, neurological development with behavior changing technique, relaxation and pain management and educational apps In addition, with this, there also several approaches through mobile apps were validated for diagnosis and daily life improvement which could be highly effective to the CP individuals and caregivers.

The studies included present a varied spectrum of technological interventions aimed at improving the lives of individuals with CP, highlighting the potential of mobile technology in addressing diverse aspects of their needs. In our included studies, most of the participants were individuals with CP [18-22]. However, the apps were built for different types of CP individuals, such as those who used sign language, had neurodevelopmental disabilities, and had different levels of motor disabilities, enhancing accessibility and communication for a broader range of individuals [18,20,21]. One study found the efficacy of technology in sports performance evaluation among CP individuals, potentially aiding in their athletic development [17]. Additionally, one study evaluated the impact of an educational mobile app on caregivers' knowledge of CP [19], while another helped in managing chronic pain in children with CP [22]. Collectively, all the findings of the included studies suggested the vital and positive impact of mobile-based interventions on supporting CP individuals and caregivers.

We additionally underlined several studies that were based on the validation of mobile apps in CP management, spanning various domains such as physical assessment, remote monitoring, self-management, and early diagnosis. The reliability of smartphone-based goniometer apps in measuring ROM, measuring migration percentage on hip surveillance radiographs in children with CP, and three-dimensional models of the femur from conventional radiographs has been highly validated in different studies [24,26,27]. In addition, capturing infant movements of high- and low-risk infants in early detection of CP, self-management, and personalized care for individuals with disabilities, including CP, early detection of CP through perinatal brain injuries also has opportunities to serve individuals with CP and CP management.

In our findings, all the studies underscored the significant role of mobile technology in addressing various facets of CP management, spanning communication, therapy, sports, education, and various domains such as physical assessment, remote monitoring, self-management, early diagnosis, and pain management. These interventions exhibited promising results, offering innovative and accessible solutions that can positively impact the lives of individuals with CP and their caregivers. Moreover, the studies emphasized the importance of tailored, user-centric design and the potential for technology to augment traditional care methods in supporting individuals with CP across different domains of their lives. Future research should prioritize well-designed randomized controlled trials with larger, diverse populations, longer follow-up, and standardized QoL measures to provide robust evidence on the effectiveness of mobile apps in CP care. Indeed, most of these studies have been conducted in HICs, revealing a technological disparity that exists in LMICs. Despite this gap, given the high prevalence and significant disease burden of conditions like CP management in LMICs, there's a pressing need to initiate the use of these technologies in these regions [3]. Innovation, government policy support could play an important role in increasing the use of technology and apps related to disabilities, including CP in LMICs [28]. Additionally, educational and capacity-building training would be helpful to understand the importance of mobile apps in healthcare professionals as well as caregivers [29]. Another important point was that caregivers of children with cerebral palsy in LMICS settings face distinctive challenges, stemming from a convergence of gender norms, poverty, stigmatization, and non-inclusive public policies [30]. Addressing these issues and supporting those caregivers through advanced technology and mobile apps is crucial to enhancing the quality of life for these caregivers.

Our study has several limitations. Firstly, the studies analyzed in this review showed significant heterogeneity and also did not report detailed participant characteristics such as Gross Motor Function Classification System (GMFCS) levels or motor types, making it impossible to conduct a meta-analysis and draw broad conclusions. Additionally, most of the included studies were rated as low or very low quality, with small sample sizes, short follow-up periods, heterogeneity in study designs and outcomes, and limited methodological rigor (lack of randomization), indicating that the evidence base for the effectiveness of mobile apps in supporting individuals with CP and their caregivers is not strong. Furthermore, the short follow-up periods in most studies limited the ability to assess long-term effectiveness and sustainability of the interventions. Lastly, the small sample sizes in several studies can limit the statistical power and generalizability of the findings, highlighting the need for larger, well-designed studies.

## Conclusions

Mobile technology holds immense potential to revolutionize the care landscape for individuals with CP and their caregivers. These interventions, tailored for various types and severities of CP, cover a wide range of domains from communication and therapy to sports, education, pain management and early detection. However, it's crucial to acknowledge the technological disparity between high-income and low- to middle-income settings, highlighting the urgency to introduce and implement these technologies in regions facing significant CP burdens. Additionally, recognizing the unique challenges faced by caregivers of children with CP in low-and middle-income settings may play an important role in supporting these caregivers through advanced technological solutions.

## Appendices

**Table 5:** Rationale of judgement of included studies on ROBIN-I assessment app: application; ROBIN-I: Risk Of Bias In Non-randomised Studies - of Interventions

Study	Bias due to confounding	Bias in selection of participants into the study	Bias in classification of interventions	Bias due to deviations from the intended intervention	Bias due to missing data	Bias in measurement of outcomes	Bias in selection of the reported result	Overall risk of bias
Zamin et al. [22]	Serious	Serious	Low	Low	Low	Moderate	Moderate	Serious
Rationale for judgement	The study does not address confounding factors or control for variables that could influence the results, such as individual differences in participants or external factors.	The selection process focuses on a single participant, significantly limiting the study's external validity and generalizability.	The intervention (the "Make Me Speak" app) is clearly defined, and there are no apparent inconsistencies in how it is applied or classified.	There is no indication of deviations from the intended intervention, and the app was used as designed.	There is no evidence of missing data, especially given the small sample size (one participant).	The outcomes are based on observational data, without objective measurement tools, which increases the risk of measurement bias.	Given the observational nature and single case, there is a risk that the study selectively reports favorable outcomes while ignoring neutral or negative results.	Due to the small sample size, lack of adjustment for confounders, and limited scope, the overall risk of bias in the study is serious.
Johnson et al. [20]	Moderate	Low	Low	Low	Moderate	Moderate	Moderate	Moderate
Rationale for judgement	The study does not explicitly account for confounding variables that could influence the effectiveness of the intervention such differences in children's disabilities or home environments, though it recognizes some of the diverse needs of the population.	Participants were selected based on clear inclusion criteria (children aged 6-12 years with neurodevelopmental disabilities) for prototype testing. While the sample size is small, recruitment seems unbiased.	The intervention with gamified therapy app is well-defined and consistently applied throughout the study, reducing the risk of misclassification.	There are no indications of significant deviations from the intended intervention. The app was tested as designed with the specified behavior change techniques (BCTs) and gamification elements.	Although the study does not mention significant missing data, the small sample size (only four children completed the user testing) limits the robustness of the findings, and missing feedback from one participant could introduce bias.	The outcomes are assessed through qualitative feedback and user experience ratings, but challenges with the "think-aloud" testing protocol due to participant limitations such as communication difficulties may introduce bias in outcome measurement.	The study reports positive results from user testing, but the small sample and qualitative nature of the findings may lead to selective reporting, as negative or neutral experiences could be underreported.	The study shows some strengths in design and implementation, but the small sample size, challenges in data collection, and qualitative nature of the outcomes introduce moderate risk of bias.
Silva et al. [18]	Moderate	Serious	Low	Low	Low	Moderate	Low	Moderate
Rationale for judgement	The study did not address potential confounders in detail. While it aimed to help individuals with speech and motor impairments using the AACVOX app, it did not consider variations in motor impairments or socioeconomic factors that might affect the accessibility or usability of the tool.	Participants (20 volunteers) were recruited from two institutions, which could introduce selection bias, as they were all boccia players. This does not represent a random or broad selection of individuals with cerebral palsy and speech impairments.	There is no indication that the classification of participants' motor impairments could have been incorrectly applied. The study clearly defines motor impairment levels based on established classifications for cerebral palsy.	The study does not mention any significant deviations from the intended intervention (use of the AACVOX app). All participants followed a predefined test protocol.	There is no mention of missing data or participants dropping out. The small sample size (20 participants) limits the generalizability but does not introduce missing data bias.	The outcomes were based on a usability questionnaire (SUS), which is subjective and could be prone to response bias, especially since participants might not want to provide negative feedback. Moreover, the use of self-report scales can lead to measurement bias if participants are not fully accustomed to using such scales.	There is no evidence that the authors selectively reported results. All tasks and SUS questionnaire results appear to be presented comprehensively.	While the study methodology is clear and follows standard protocols, issues with participant selection, potential confounding, and subjective outcome measures (self-reported usability) limit the strength of the findings. However, there is no indication of severe flaws or missing data that would suggest a serious or critical risk of bias.
Coswig et al. [17]	Moderate	Moderate	Low	Low	Low	Moderate	Low	Moderate
Rationale for judgement	The article clearly identifies the focus on CP football players and controls variables like prior experience with jump exercises, but does not fully consider other potential confounding variables, such as the influence of different neurologic impairments.	Participants were selected from a specific competition (Brazilian CP Football Championship). While appropriate for the study, this could introduce selection bias as it does not represent all CP football players.	The interventions (MyJump2 app vs. contact mat) were clearly classified and applied consistently across participants.	There were no deviations from the planned intervention (use of MyJump2 app), and the protocol was followed throughout the study.	There is no indication of missing data or participant dropouts, with all 40 participants completing the jump tests.	The outcome measures (jump height and flight time) were objectively assessed using two instruments (MyJump2 app and contact mat). However, the reliance on a single evaluator for app measurements could introduce some measurement bias.	Results were comprehensively reported with no indication of selective reporting. The data were presented for both the MyJump2 app and the contact mat, with strong statistical support.	While the study is well-conducted, potential biases arise from participant selection and some aspects of outcome measurement, but these do not significantly undermine the validity of the findings.
Ghazisaeedi et al. [19]	Moderate	Serious	Low	Low	Low	Moderate	Low	Moderate
Rationale for judgement	The study does not fully account for potential confounders, such as variations in the caregivers' prior knowledge or differences in the severity of the children's conditions.	Caregivers were recruited from a single occupational therapy center and through convenience sampling, which may introduce selection bias and limit generalizability.	The intervention (use of the Android application) was consistently applied to all participants. No concerns about misclassification were noted.	There were no significant deviations from the intended use of the application, and all participants followed the same protocol.	Three caregivers dropped out of the study, but the remaining data were analyzed for all other participants, reducing the risk of bias due to missing data.	The outcomes were self-reported, which could introduce measurement bias, especially in areas where caregivers may have overestimated their knowledge or familiarity with the application.	The results were comprehensively reported, and there was no evidence of selective reporting or omission of negative findings.	The study is well-conducted, though limited by participant selection and potential confounders, especially caregivers' prior knowledge. Consistent intervention and detailed reporting reduce major bias.
Ostojic et al. [22]	Moderate	Serious	Low	Moderate	Serious	Serious	Low	Serious
Rationale for judgement	While the study acknowledges potential confounders such as co-occurring anxiety and prior treatments, these were not fully controlled. The lack of clear management of these variables introduces a moderate risk of bias.	Participants were recruited from specific hospitals and registries, and children with moderate-to-severe intellectual impairments were excluded. This non-randomized recruitment and exclusion of certain groups could introduce significant bias and limit the generalizability of the findings.	The intervention (BrightHearts app) was delivered consistently across participants with no deviations. All participants received the same training and were instructed similarly, so there is a low risk of bias in this domain.	Some participants faced challenges with regular app use due to time constraints, attention issues, and boredom, resulting in inconsistent adherence. While these reflect real-world challenges, they may moderately impact outcomes.	Missing post-intervention data (e.g., quality of life and anxiety surveys) for some participants is a significant issue, especially in a small sample size. This reduces the reliability of the study’s conclusions and represents a serious risk of bias.	The study relied on self-reported measures of pain and anxiety, which are subjective and prone to bias. Additionally, no blinding was implemented, increasing the risk of biased outcome reporting.	Both qualitative and quantitative findings were reported, including non-significant results, suggesting no selective outcome reporting. Therefore, this domain has a low risk of bias.	Due to participant selection issues, missing data, and reliance on subjective, self-reported outcomes without blinding. These factors undermine the study’s ability to draw definitive conclusions.
